# Oligohydramnios in a pregnant Pakistani woman with *Plasmodium vivax* malaria

**DOI:** 10.1186/1475-2875-13-156

**Published:** 2014-04-23

**Authors:** Nicolò Binello, Enrico Brunetti, Federico Cattaneo, Raffaella Lissandrin, Antonello Malfitano

**Affiliations:** 1 Division of Infectious and Tropical Diseases, San Matteo Hospital Foundation, University of Pavia, 19, V. le Golgi, Pavia 27100, Italy

**Keywords:** *Plasmodium vivax*, Malaria, Pregnancy, Oligohydramnios, Relapsing malaria, Pre-term birth

## Abstract

In the Western world, the diagnosis and management of *Plasmodium vivax* malaria in pregnant women can be challenging, and the pathogenesis of adverse outcomes for both the mother and the foetus is still poorly known. The authors describe the case of a 29-year-old Pakistani woman at the 29^th^ week of her second pregnancy, who was admitted to the Hospital following the abrupt onset of fever. At the time of admission, she had been living in Italy without travelling to any malaria-endemic areas for eight months. She was diagnosed with vivax malaria after a thin blood smear revealed the presence of plasmodial trophozoites and gametocytes and treated accordingly. Due to the onset of oligohydramnios, she underwent caesarian section at the 31^st^ week of pregnancy with no further complications. Histological examination of the placenta showed no evidence of plasmodial infection, but was inconclusive. It is unclear whether oligohydramnios is a complication of pregnancy-related *Plasmodium vivax* malaria. Given the long latency of hypnozoites, every febrile pregnant patient with a previous stay in an endemic area should be screened for malaria with a thick and a thin blood smear.

## Background

Every year approximately 50 million women living in malaria-endemic areas become pregnant [1]: it is estimated that 10,000 women and 200,000 infants die as a result of malaria complications during pregnancy. *Plasmodium falciparum* is the most frequent cause of severe clinical manifestations, including maternal anaemia, prematurity, and low birth weight. However, *Plasmodium vivax* malaria can also cause some of the same complications as *P. falciparum* infection, although less commonly.

Most clinical manifestations are nonspecific and variable. Fever is the main sign, usually accompanied by chills and sweats. Other common symptoms include headache, myalgias, fatigue, nausea and vomiting, abdominal pain and diarrhoea. Pregnant women are more susceptible than non-pregnant women to develop complications, such as hypoglycaemia, pulmonary oedema and acute respiratory distress syndrome [2]. Approximately 60 percent of pregnant women are anaemic as a result of malarial infection [3,4], and anaemia may be the only finding of the disease [5]. Malaria during pregnancy can result in a number of adverse outcomes in the newborn, including miscarriage, foetal growth restriction/small for gestational age (SGA) infant, preterm birth (<37 weeks of gestation), low birth weight (LBW) (<2500 g at birth), congenital and post-partum infection, and perinatal death [3,6-9].

## Case presentation

A 29-year-old Pakistani woman, gravida 2 para 1, at the 29^th^ + 6 week of her second pregnancy, was admitted to the Emergency Room (E.R.) of San Matteo Hospital Foundation (Pavia, Italy) on the 30^th^ of May 2012 following the abrupt onset of fever and malaise and was then transferred to the Obstetrics and Gynecology (OB/GYN) Department.

The woman was born and had always lived in Pakistan until eight months before admission when she moved to Italy and had been living there thenceforth. She reported having been well until the day before admission. She denied any history of previous disease. Her first pregnancy was uncomplicated, and she delivered a healthy newborn at the 40^th^ week. On entrance, she was febrile (38°C). Her physical examination was unremarkable. Blood pressure was 120/70 mmHg and the remainder of her vital signs were normal.

Routine blood tests showed a haemoglobin (Hb) level of 11.5 g/dl, 33.7% haematocrit (Hct) and a mild neutrophilic leukocytosis (WBC 10.6 × 10^9^/L: neutrophils 8.2 × 10^9^/L). She was started on ampicillin (1 g tid) and paracetamol. Fever subsided, and cardiotocography (CTG) did not reveal any signs of foetal distress. Three days after the beginning of treatment, fever with shivers recurred in association with lumbar and leg pain. Gentamicin (80 mg tid) was added, and then, on the basis of the Infectous Disease (I.D.) consult, antibiotic therapy was switched into piperacillin/tazobactam (4.5 g tid) in order to cover a possible urinary tract infection. The patient remained afebrile for one day, when blood tests showed an increase of bilirubin (total 2.80 mg/dl; direct 1.48 mg/dl) and a decrease of haemoglobin (Hb 10.8 g/dl, Hct 32.7%). The next day the temperature rose to 39.8°C, the haemoglobin value dropped to 10.3 g/dl, and an abnormal foetal heart rate variability was recorded on the CTG. In order to rule out pneumonia, a chest X-ray was performed under a protective abdominal shield and resulted negative. A hematological consult was obtained, too, and a thin blood smear was examined, detecting the presence of *Plasmodium spp*. The I.D. specialist identified trophozoites and gametocytes from *Plasmodium vivax*, thus enabling the diagnosis of *P. vivax* pregnancy-associated malaria. Quinine sulphate (500 mg tid) was immediately administered, and therapy was continued for seven days.

During malaria treatment, the patient remained afebrile and asymptomatic. One week after the initiation of therapy, the haemoglobin level increased to 11.8 g/dl and bilirubinaemia returned to normal values (total 0.95 mg/dl). At the same time (31^st^ week), an ultrasound exam showed the presence of oligohydramnios, and the amniotic fluid index (AFI) was 16 mm. As a matter of fact, four previous ultrasound exams, the first two preceding quinine administration, had shown a progressive decrease of the amniotic fluid index. The patient underwent a premature rupture of membranes (PROM) test, which was negative, and a caesarean section followed, with no further complications.

Thin blood smears were obtained from both umbilical and peripheral blood of the newborn, tested for malaria parasites, and resulted negative. Concurrently, the antigen test (Alere BinaxNOW®, Milan, Italy) was performed on the umbilical blood and turned out to be negative. Furthermore, the histological exam of the placenta was requested, but no pathological evidence of infection was observed. The patient was then discharged, and a follow-up appointment was scheduled to initiate the eradication therapy through the long-term administration of primaquine.

## Discussion

Infection from *P. vivax* in pregnancy has traditionally been considered less severe as compared to *P. falciparum* malaria. This is believed to be related to the lack of placental sequestration in *P. vivax* infection and the parasite tropism for reticulocytes accounting for a milder form of anaemia [10,11]. The patient’s lowest haemoglobin level was 10.3 g/dl. Nevertheless, especially in undiagnosed patients, pregnancy-related *P. vivax* infection correlates with a significant risk of adverse maternal and foetal outcomes, and a prompt diagnosis is of paramount importance to assess the prognosis *quoad vitam* and *quoad valetudinem* of both the foetus and the mother [12-14]. This case highlights the major issues of the diagnostic process and the clinical management of *P. vivax* infection in pregnant women.

The diagnosis of malaria was made only one week after admission. The patient was born in Pakistan but she had not travelled to any malaria endemic areas for nearly one year. Hence, the correlation between the onset of fever and other nonspecific symptoms and malarial infection was not promptly considered. Lumbar pain and intermittent fever, despite the initiation of ampicillin therapy, skewed the differential diagnosis toward a urinary tract infection. Nonetheless, the diagnosis of pregnancy-associated malaria could not be excluded. Indeed, *P. vivax* and *Plasmodium ovale* infections may result in a long period of clinical latency due to the presence of hepatic hypnozoites, which can re-activate weeks, or even months, after the exposure to the parasite. In this case, the onset of symptoms was eventually demonstrated to be caused by *P. vivax* malaria nearly one year after the last exposure. Thus, the suspicion of pregnancy-associated malaria should be elicited in any febrile pregnant woman with a previous stay in a malarious region and malaria screening tests should be always performed.

A second major issue of pregnancy-related malaria is therapy. First-line therapy of uncomplicated *P. vivax* malaria is chloroquine. Nevertheless, several cases have been reported describing the presence of chloroquine-resistant *P. vivax* isolates in a number of areas throughout Asia and Oceania, including the Indian subocontinent [15,16]. In addition, a mixed infection could not be ruled out. Indeed, *P. falciparum* can account for mild cases of malaria in pregnant women who left an endemic country, because the reduction of immunity related to pregnancy might result in a recrudescence of low-level parasitaemia even several months later [17,18]. Accordingly, the I.D. specialist chose quinine sulphate as elective treatment. Moreover, in order to prevent *P. vivax* relapsing infection by eradicating the dormant forms, primaquine constitutes the standard therapy. However, because primaquine can induce haemolytic anaemia in persons affected by G6PD deficiency, it is contraindicated in pregnant women given the unknown neonatal G6PD status [19]. Hence, the patient started primaquine treatment after delivery.

Finally, the presence of malarial infection in the newborn must be assessed immediately at birth. Thin blood smears were performed on the umbilical and peripheral blood, detecting no parasites. Search for plasmodial antigens was negative, too. Nonetheless, it is unclear whether the oligohydramnios was a foetal adverse outcome of malaria or a pregnancy complication unrelated to the maternal infection. The histological examination of the placenta is of great significance for the diagnosis of *P. falciparum*, may be useful as a confirmation test in non-falciparum malaria, and may be predictive of adverse outcomes in the newborn [20,21]. Placental histology was investigated and revealed no sign of infection. However, unlike *P. falciparum*, *P. vivax* rarely correlates with pathological evidence of infection, due to its typical lack of cytoadherence to placenta [10], and a negative result of the histological exam does not rule out the presence of *P. vivax* within the placenta. In addition, the correlation between maternal infection and adverse outcomes in the newborn needs further investigation, and the effects of *P. vivax* malaria during pregnancy have not been fully characterized. Therefore, the progressive contraction of the amniotic fluid might be explained by an ongoing *P. vivax* infection, which was not detected by placental examination. Lastly, since the amniotic fluid index started decreasing before the initiation of therapy, the quinine treatment was unlikely to account for the oligohydramnios. No other maternal or placental causes of oligo-anhydramnios were found.

## Conclusions

The pathogenesis of adverse outcomes in pregnancy-related *Plasmodium vivax* malaria is still poorly known, and the management of malaria in pregnant women can be challenging. Every febrile or anaemic pregnant patient with a previous stay in an endemic area should be screened for malaria. The long latency of *P. vivax* malaria dictates the inclusion of such infection in the differential diagnosis, in so far as the suspicion is supported by epidemiological data. Placental examination should be recommended upon delivery: evidence of malarial infection might provide useful insights on the pathogenesis of congenital malaria as well as the occurrence of potential complications in the newborn. However, a negative result does not exclude an active non-falciparum malarial infection. Future studies should investigate the link between oligo-anhydramnios and pregnancy-associated *P. vivax* malaria.

## Consent

Written informed consent was obtained from the patient for publication of this case report and any accompanying images. A copy of the written consent is available for review by the editor-in-chief of this journal.

## Abbreviations

AFI: Amniotic fluid index; E.R.: Emergency room; I.D.: Infectious diseases; Hb: Haemoglobin; Hct: Haematocrit; LBW: Low birth weight; OB/GYN: Obstetrics and gynecology; PROM: Premature rupture of membranes; SGA: Small for gestational age; WBC: White blood cells.

## Competing interests

The authors declare that they have no competing interests.

## Authors’ contributions

NB, EB, AM drafted the paper, NB, RL, FC collected the data. All authors discussed and approved the final version of the paper.

## References

[B1] Schantz-Dunn J Nawal MN Malaria and pregancy: a global health perspective Rev Obstet Gynecol 2009 Summer, 2 3 186 192 19826576 PMC2760896

[B2] World Health Organization Division of control of tropical diseases: severe and complicated malaria Trans R Soc Trop Med Hyg 1990 84 Suppl 2 1 65 2219249

[B3] Espinoza E Hidalgo L Chedraui P The effect of malarial infection on maternal-fetal outcome in Ecuador J Matern Fetal Neonatal Med 2005 18 101 105 10.1080/147670500231989 16203594

[B4] Assabri AM Muharram AA Malaria in pregnancy in Hodiedah, Republic of Yemen East Mediterr Health J 2002 8 245 253 15339111

[B5] Whitty CJ Edmonds S Mutabingwa TK Malaria in pregnancy BJOG 2005 112 1189 1195 10.1111/j.1471-0528.2005.00714.x 16101595

[B6] Hulbert TV Congenital malaria in the United States: report of a case and review Clin Infect Dis 1992 14 922 926 10.1093/clinids/14.4.922 1576289

[B7] McGready R Lee SJ Wiladphaingem J Ashley EA Rijken MJ Boel M Simpson JA Paw MK Pimanpanarak M Mu O Singhasivanon P White NJ Nosten FH Adverse effects of falciparum and vivax malaria and the safety of antimalarial treatment in early pregnancy: a population-based study Lancet Infect Dis 2012 12 388 396 10.1016/S1473-3099(11)70339-5 22169409 PMC3346948

[B8] Newman RD Hailemariam A Jimma D Dedifie A Kebede D Rietveld AEC Nahlen BL Barnwell JW Steketee RW Parise ME Burden of malaria during pregnancy in areas of stable and unstable transmission in Ethiopia during a nonepidemic year J Infect Dis 2003 188 1259 1261 10.1086/378678 12751034

[B9] Carles G Bousquet F Raynal P Peneau C Mignot V Arbeille P [Pregnancy and malaria. Study of 143 cases in French Guyana](in French) J Gynecol Obstet Biol Reprod (Paris) 1998 27 798 805 10021993

[B10] McGready R Davison BB Stepniewska K Cho T Shee H Brockman A Udomsangpetch R Looareesuwan S White NJ Meshnick SR Nosten F The effects of *Plasmodium falciparum* and P. vivax infections on placental histopathology in an area of low malaria transmission Am J Trop Med Hyg 2004 70 398 407 15100454

[B11] Mayor A Bardají A Felger I King CL Cisteró P Dobaño C Stanisic DI Siba P Wahlgren M del Portillo H Mueller I Menéndez C Ordi J Rogerson S Placental infection with *Plasmodium vivax:* a histopathological and molecular study J Infect Dis 2012 206 1904 1910 10.1093/infdis/jis614 23053630 PMC6592414

[B12] Nosten F McGready R Simpson JA Thwau KL Balkan S Cho T Hkirijaroen L Looareesuwan S White NJ Effects of *Plasmodium vivax* malaria in pregnancy Lancet 1999 354 546 549 10.1016/S0140-6736(98)09247-2 10470698

[B13] Poespoprodjo JR Fobia W Kenangalem E Lampah DA Warikar N Seal A McGready R Sugiarto P Tjitra E Anstey NM Price RN Adverse pregnancy outcomes in an area where multidrug-resistant *Plasmodium vivax* and *Plasmodium falciparum* infections are endemic Clin Infect Dis 2008 46 1374 1381 10.1086/586743 18419439 PMC2875100

[B14] Singh N Shukla MM Sharma VP Epidemiology of malaria in pregnancy in central India Bull World Health Organ 1999 77 567 572 10444880 PMC2557706

[B15] Garg M Gopinathan N Bodhe P Kshirsagar NA Vivax malaria resistant to chloroquine: case reports from Bombay Trans R Soc Trop Med Hyg 1995 89 656 657 10.1016/0035-9203(95)90432-8 8594687

[B16] Van Den Abbeele K Van Den Enden E Van Den Ende J Combined chloroquine and primaquine resistant *Plasmodium vivax* malaria in a patient returning from India Ann Soc Belge Med Trop 1995 75 73 74 7794065

[B17] Kantele A Siikamäki H Hannila-Handelberg T Laitinen K Rombo L *Plasmodium falciparum* - malaria in pregnant African immigrants often goes unrecognized J Travel Med 2012 19 380 382 10.1111/j.1708-8305.2012.00651.x 23379709

[B18] Odolini S Apostoli A Casari S Matteelli A Castelli F Recrudescence of *Plasmodium falciparum* malaria in a primigravid woman with anaemia as the only sign of disease J Obstet Gynaecol 2014 [Epub ahead of print]. doi:10.3109/01443615.2013.866079 10.3109/01443615.2013.866079 24484295

[B19] Hollier LM Ericksen AL Cox SM Malaria in pregnancy Infect Dis Obstet Gynecol 1997 5 45 51 10.1155/S1064744997000100 18476133 PMC2364524

[B20] Mockenhaupt FP Ulmen U von Gaertner C Bedu-Addo G Bienzle U Diagnosis of placental malaria J Clin Microbiol 2002 40 306 308 10.1128/JCM.40.1.306-308.2002 11773140 PMC120131

[B21] Smereck J Malaria in pregnancy: update on emergency management J Emerg Med 2011 40 393 396 10.1016/j.jemermed.2010.04.029 20566259

